# Effect of Aspirin Versus Clopidogrel on Walking Exercise Performance in Intermittent Claudication—A Double-Blind Randomized Multicenter Trial

**DOI:** 10.1161/JAHA.111.000067

**Published:** 2012-02-20

**Authors:** Elisabeth Singer, Stephan Imfeld, Daniel Staub, Ulrich Hoffmann, Ivo Buschmann, Karl-Heinz Labs, Kurt A. Jaeger

**Affiliations:** Department of Angiology, University Hospital, University of Basel, Switzerland (E.S., S.I., D.S., K.-H.L., K.A.J.); Department of Angiology, Medical Policlinic, University Hospital, Munich, Germany (U.H.); Centre for Cardiovascular Research and Experimental and Clinical Research Centre (ECRC), Charité, Berlin, Germany (I.B.)

**Keywords:** antiplatelet therapy, claudication, exercise training, peripheral artery disease

## Abstract

**Background:**

This study sought to determine possible effects of different antiplatelet therapies on walking exercise performance in intermittent claudication. Aspirin, in contrast to clopidogrel, interferes with processes that increase collateral conductance in an ischemic animal model.

**Methods and Results:**

Patients with stable intermittent claudication were recruited from 21 centers in Switzerland and Germany and randomized to either aspirin or clopidogrel treatment. They participated in a 3-month rehabilitation program (electronically monitored, home-based, 1-hour daily walking sessions at a speed of approximately 120 steps/min). Walking distance was assessed by treadmill tests (3.2 km/h; 12% grade) at baseline and after 12 weeks. A total of 229 of 259 patients with a mean age of 66.2±7.7 years completed the study according to the protocol. A total of 24.5% were females, 20.1% diabetics, and 85.6% were active/ex-smokers. The baseline characteristics were a median (interquartile range) ankle/brachial index of 0.69 (0.57±0.8), an initial claudication distance (ICD) of 98 m (70 to 151 m), and an absolute claudication distance (ACD) of 162 m (113 to 302 m). Training resulted in a median increase of initial claudication distance by 33.5 m (33.3%) in the clopidogrel group and 29 m (33.9%) in the aspirin group. The values for absolute claudication distance were 60.5 m (34.9%) and 75 m (35.3%), respectively (p_ICD_=0.42 and p_ACD_=0.66).

**Conclusions:**

Treatment with aspirin did not show a difference in initial claudication distance or absolute claudication distance improvements compared with clopidogrel after a 3-month walking rehabilitation program. **(*J Am Heart Assoc*. 2012;1:51-56.)**

**Clinical Trial Registration:**

URL: http://www.ClinicalTrials.gov. Unique identifier: NCT00189618, URL: https://EudraCT.ema.europa.eu, Unique identifier: 2004-005041-35

Walking exercise and antiplatelet drugs are the first-line therapy in patients with intermittent claudication.^1^ With respect to vascular morbidity and mortality, clopidogrel is more effective in patients with symptomatic peripheral artery disease (PAD) compared with aspirin.^2^ Walking exercise increases the pain-free walking distance by improving gait economy and collateralization. The latter process, called arteriogenesis, is an important mechanism in both cardiac and peripheral ischemic events and has been investigated intensively for pharmacological stimulation in experimental settings. So far, none of the larger trials have shown clinical benefits in peak walking time in PAD patients.^3–5^ Arteriogenesis is driven by shear–stress induced remodeling of preexisting small arteries mediated by a localized inflammatory reaction. Expression and presentation of adhesion factors as well as upregulation of chemokines and colony-stimulating factors by the endothelium attract monocytes. After transmigration and maturation to macrophages, these cells maintain the localized inflammation that allows for the remodeling process.^6^ As shown recently in an ischemic animal model by Hoefer et al, aspirin, due to its antiinflammatory properties, inhibits this localized inflammation, whereas clopidogrel does not, resulting in decreased vascular collateral conductance in animals treated with aspirin.^7^ There are also some preliminary indications that patients who responded well to supervised exercise training may have an additional improvement in walking distance if they were treated with clopidogrel as compared with aspirin.^8^

On the basis of these findings,^6,7^ a double-blind randomized trial was conducted, investigating the hypothesis that aspirin treatment may lead to a smaller training-associated improvement of walking distance following a 3-month exercise program compared with clopidogrel.

## Methods

### Patients

Patients with known peripheral artery disease were recruited from 21 Swiss and German outpatient centers. Requirements for inclusion were Fontaine stage II claudication with a resting ankle/brachial index of <0.95, pain-free claudication distance between 50 and 400 m, constant management of concomitant diseases, and a history of stable claudication for at least 3 months. Patients were excluded if they required oral anticoagulants or underwent surgical or catheter-directed revascularization within the last 3 months. Further exclusion criteria were age below 45 years, diagnosis of thrombangitis obliterans, severe peripheral neuropathy (defined as sensibility to vibration <4/8 and/or absence of Achilles tendon reflexes), comorbid conditions interfering with walking exercise, and a life expectancy of ≤2 years. Patients with known intolerance to aspirin or clopidogrel, regular intake of non-steroidal antiinflammatory drugs, diagnosis of peptic ulcer, or gastrointestinal bleeding within the previous 6 months were also excluded. Written informed consent was obtained from all patients.

Patients were only eligible to participate if 2 treadmill tests conducted within 3 to 5 days showed initial claudication distance (ICD) values differing ≤25%. Eligible patients were randomized stratified by site using block randomization with blocks of 2 in a double-blind fashion to aspirin 100 mg/day or clopidogrel 75 mg/day and entered a home-based rehabilitation program for 12 weeks.

### Exercise Training and Monitoring

Exercise consisted of daily 1-hour walking sessions. Patients were equipped with a metronome (QT-5™, Evets Corp, San Clemente, CA) and instructed to walk at a speed of 120 single steps per minute. Training sessions were monitored using an electronic exercise-recording device (PACER™, FitSense Technologies Inc, Southborough, MA) to check the compliance with training requirements. During biweekly visits, data from the recording devices were downloaded and checked for training compliance. Advice to improve training was given if necessary. Adherence to the study drugs was monitored by pill count.

### Measurements

Walking distances (ICD and absolute claudication distance [ACD]) were recorded by constant load treadmill testing at baseline and after 12 weeks (standard settings of 3.2 km/h, 12% slope). Duplex sonography from the common iliac to the popliteal arteries was performed on both legs at baseline. Ankle brachial index was calculated for each leg separately by dividing the higher of the anterior and posterior tibial artery systolic pressures by the higher of the systolic left and right brachial pressures. Primary and secondary end points were percent change in ICD and ACD improvement, respectively.

### Statistics

The sample size was calculated on the basis of a previous study^8^ with an estimated group difference of 30%, a standard deviation of 80%, and a power of 80% using a 2 independent sample *t*-test with a 2-sided level of significance of 5%. The total number of patients required was thus estimated to be 112 patients per treatment arm plus 20% to compensate for the estimated drop out rate.

Statistical Analysis was performed by an independent biometrics company (Koehler GmbH, Freiburg, Germany) using SAS-Software version 9.1. As the primary intention was to investigate the presumed mechanism of arteriogenesis interference of aspirin as compared with clopidogrel, results of the per-protocol analysis are being presented.

Data did not show normal distributions; hence, nonparametric tests and descriptives were used throughout the study. Wilcoxon rank sum tests were performed to test for differences in percent change between groups. An analysis of covariances based on ranks was used to investigate possible confounding factors. All values represented herein are *medians* and *interquartile ranges. P* values of ≤0.05 were considered statistically significant.

The study was approved by the German and Swiss national competent authorities and ethics committees.

## Results

**Figure 1.**
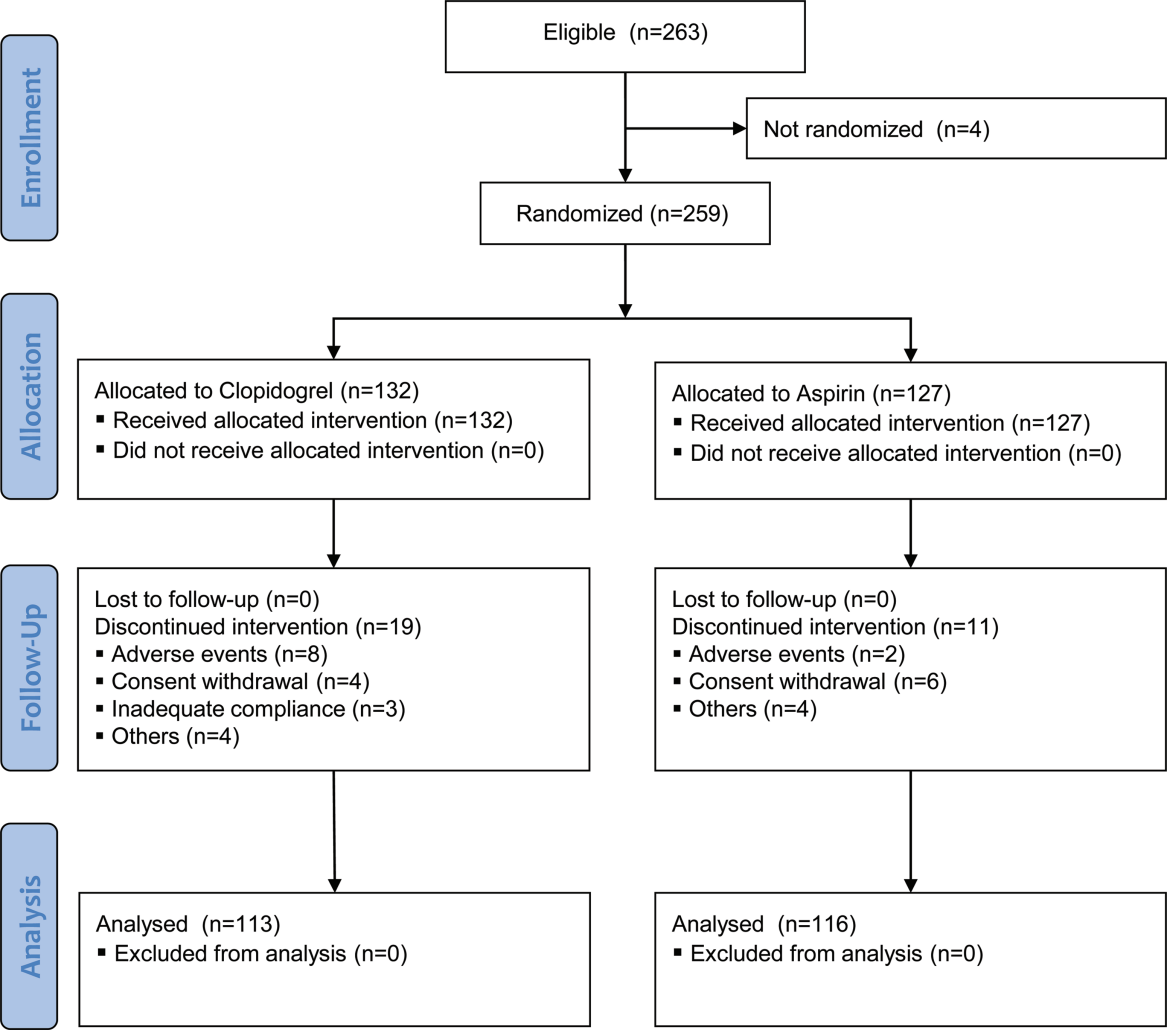
Consolidated Standards of Reporting Trials (CONSORT) flow diagram of patients through each stage of the trial.

The detailed trial profile is shown in Figure 1. Of the 259 claudicants randomized, 229 completed the full course of the study, 116 randomized to aspirin and 113 to clopidogrel, resulting in a drop out rate of 11.6%. There were no serious adverse events. Reasons for dropout were unwillingness to further participate in 9 cases, disease progression in 3 cases, malcompliance with exercise training in 3 cases, skin reaction to study drug (clopidogrel) in 3 cases, worsening claudication of nonvascular origin in 3 cases, alcohol withdrawal in 2 cases, elevated liver enzymes (>3-fold upper limit), worsening of epistaxis, pancreatitis, stenocardia, protocol violation, inaccurate randomization, and personal reasons each in 1 case. Baseline characteristics are listed in Table 1.

**Table 1.** Baseline Characteristics of Subjects Randomized to Aspirin or Clopidogrel

	Aspirin N=116	Clopidogrel N=113
Median age (years) (IQR)	66 (60–71.5)	66 (61–72)

Women	29.3	19.5

Median ICD (meter) (IQR)	98 (69–145)	89 (67–169)

Median ACD (meter) (IQR)	160 (112–294)	168 (111–341)

Median ABI (IQR)	0.65 (0.56–0.82)	0.73 (0.61–0.81)

Location of main arterial obstruction		

Iliac	13.3	21.6

Femoro-popliteal	80.5	70.7

Distal	5.3	6.9

Tobacco abuse		

Active smokers	37.9	34.5

Ex-smokers	45.7	53.1

Never smoked	16.4	12.4

Diabetes	23.3	16.8

Type 2	19.0	15.0

Type 1	4.3	1.8

Hypertension	26.7	17.7

Dyslipidemia	71.6	73.5

Median body mass index (IQR)	26.8 (23.9–30.12)	26.4 (24.4–28.4)

Chronic obstructive pulmonary disease	6.0	5.3

History of myocardial infarction	12.9	12.4

History of stroke	4.3	12.4

Previous peripheral revascularization (interventional/surgical)	45.7	41.6

IQR, interquartile range; ICD, initial claudication distance; ACD, absolute claudication distance; ABI, ankle brachial index. Data are expressed as percentages unless otherwise stated.

**Figure 2.**
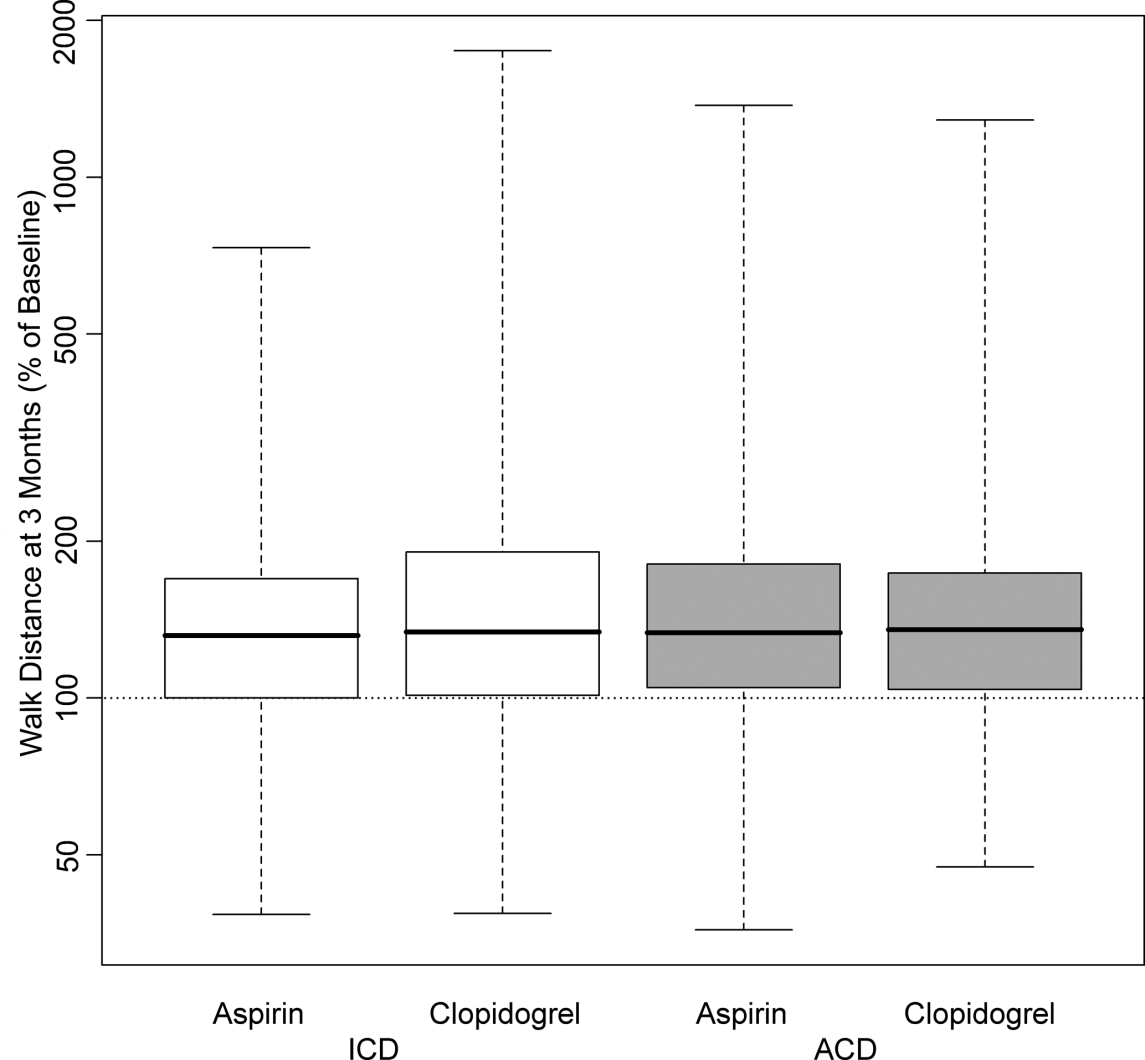
Boxplot for initial (ICD) and absolute (ACD) claudication percent change for both patient groups under aspirin and clopidogrel after 3 months training. There are no significant differences between the treatment group improvements. ICD indicates initial claudication distance; ACD, absolute claudication distance.

The median ICD increase between baseline and week 12 was 33.5 m (1 to 76 m) corresponding to an improvement of 33.9% (1.3 to 90.7%) in the aspirin group (*P*<0.001). In the clopidogrel group the median increase in ICD was 29 m (0 to 85 m) corresponding to 33.3% (0 to 70.2%, *P*<0.001). The difference between the 2 groups was not statistically significant (Figure 2, *P*=0.7 for absolute and *P*=0.42 for percentage change, respectively).

The median ACD increase between baseline and week 12 was 60.5 m (3.5 to 162 m) corresponding to 35.1% (3.9% to 71.7%) in the aspirin group. The difference in ACD of the clopidogrel group was 75 m (9 to 200 m) corresponding to 34.9% (7.7% to 81.8%). The difference between the 2 groups was also not statistically significant (*P*=0.45 and *P*=0.65, respectively). Results on group differences were unchanged when adjusted for possible confounding factors (*P*=0.20 for ICD, *P*=0.66 for ACD).

## Discussion

To our knowledge, this is the first study addressing the question whether the type of antiplatelet drug has an impact on the capacity to increase walking distance during exercise training in PAD patients with intermittent claudication. It is also one of the largest, if not the largest, randomized trial performed on walking exercise in intermittent claudication to date. Overall, the monitored home-based walking exercise led to a significant median improvement in pain-free and maximum walking distance after a 3-month training period with a surprisingly low drop out rate of 11.6%. Despite consistent experimental data^6,7^ showing that aspirin interferes with processes increasing vascular collateral conductance, the type of antiplatelet drug (aspirin or clopidogrel) had no significant influence on the improvement in walking distances in this double-blind randomized clinical trial. A possible influence on the collateral conductance itself cannot be excluded as it was not directly measured in this trial. The majority of studies focusing on aspirin and clopidogrel treatment in PAD patients assessed the drugs’ influence on reducing mortality and morbidity in the course of secondary prophylaxis.^2,9–11^ A subgroup analysis of the CAPRIE study demonstrated that among the 3 major atherosclerotic areas of cardiac, cerebrovascular, and peripheral artery disease, PAD patients showed the highest benefit from a medication with clopidogrel compared with aspirin, though the distinct mechanism of action has not yet been fully understood.^12^

Translating results from an animal experiment to a clinical application in humans is often challenging. One possible explanation for the negative results of this trial may be the dosage of aspirin. It cannot be excluded that aspirin doses in the animal model were comparatively higher with a possibly higher antiinflammatory effect than with low-dose aspirin in humans. Additionally, differences in adaptive mechanisms between the relatively acute ischemic model and the chronic nature of peripheral artery disease must be hypothesized.

Although the trial is one of the largest in PAD exercise training to date, it cannot be generally excluded that the sample size was too small to detect smaller (and therefore clinically less important) effects than anticipated. Another potential explanation for the comparable results in both groups might be the influence of training-induced changes in muscle structure and gait, which are known phenomena in PAD patients.^13,14^ Research on calf muscle characteristics showed a correlation between changes in muscle composition and lower extremity functioning.^15,16^ Capillary density of the muscle has been described to rise after 24 weeks of training.^15^ Patients in the present study were trained for only 12 weeks, as recommended by guidelines. Thus, time to effect for a benefit due to arteriogenesis, measurable by means of treadmill testing, might not have been reached. Gait normalization and training-associated muscle changes in humans with longstanding vascular disease could also have a larger effect and thus might be masking the proposed effect of antiplatelet drugs on arteriogenesis demonstrated in animals.

In terms of walking distance improvements, supervised training seems to achieve better results compared with home-based training programs, but costs are considerably higher, even in short-term programs.^17^ Recently, a randomized controlled trial was able to show that a home-based exercise program quantified with a step activity monitor achieved high adherence and was efficacious in improving claudication measures similar to a standard supervised exercise training.^18^ The patients in our study wore a pedometer to monitor their walking exercise compliance and were seen every 2 weeks to receive instructions and motivation for further training, whereas the training itself was set up clearly as a home-based exercise modality. The decision for a home-based training program was primarily due to feasibility reasons, because the institutional infrastructure for supervised training programs is often not available, and would thus have hampered a broader application of the study results. Additionally, reimbursement of supervised training by insurance companies is cut back or even lacking in many countries, which in fact often results in home-based training settings as the only alternative available. However, further research principally considering the long-term (ie, live term) effects and financial implications of supervised and home-based walking exercise is still needed for a final decision on superiority of rehabilitation programs.^8,19,20^

Previous reports on exercise training in intermittent claudication show a large heterogeneity in effect sizes, partly due to very small sample sizes. We emphasize that our reported increase of 33.9% in ICD and 33.3% in ACD are median values. Values for mean increase (as reported by most previous studies) in ICD would be 58.3% (65.3 m) and 68.1% (149.5 m) for ACD, values close to those reported by a recent Cochrane review (ICD 74.8 m, ACD 116 m) for trials reporting distance increases in supervised exercise training.^21^ This might be an indication that part of the effect of direct supervision could also be achieved by a more cost-effective indirect electronic monitoring of training activity as demonstrated in our cohort.

## Conclusions

Treatment with aspirin did not show a difference in ICD or ACD improvements compared with clopidogrel after a 3-month walking exercise training. It seems that the antiinflammatory properties of low-dose aspirin and its inhibiting effects on arteriogenesis are not of clinical relevance for rehabilitation programs in intermittent claudication.

## Appendix

List of participating study centers (in alphabetical order).

## Germany

Berlin, Evangelisches Krankenhaus Hubertus, Dr C. Fahrig

Berlin, Praxis für Gefässmedizin, Dr D. Schulte

Darmstadt, Städtische Kliniken Darmstadt, Prof Dr Bauersachs

Dresden, Universitätsklinikum Dresden, Prof Dr S. Schellong

Frankfurt, Klinikum der J.W. Goethe-Universität, Prof Dr E. Lindhoff-Last

Karlsbad, Klinikum Karlsbad-Langensteinbach, Prof Dr C. Diehm

Mainz, Johannes Gutenberg Universität, Prof Dr C. Espinola-Klein

München, Klinikum Innenstadt der Ludwig Maximilians Universität, Prof Dr U. Hoffmann

München, Praxis im Tal, Dr P. Bilderling

## Switzerland

Basel, University Hospital Basel, Prof Dr K.A. Jäger

Bellinzona, Primario Ospedale San Giovanni, Prof Dr A. Gallino

Bern, Inselspital, Prof Dr I. Baumgartner

Bruderholz, Kantonsspital Bruderholz, PD Dr C. Jeanneret

Frauenfeld, Kantonsspital Frauenfeld, Prof Dr B. Frauchiger

Lausanne, Centre Hospitalier Universitaire Vaudois, Prof Dr D. Hayoz

Liestal, Kantonsspital Liestal, Dr P. Nussbaumer

Locarno, Ospedale La Carità, PD Dr H. Stricker

Luzern, Kantonsspital Luzern, Dr M. Banyai

St. Gallen, Kantonsspital St. Gallen, Dr D. Holtz

Zürich, Universitätsspital Zürich, Prof Dr B. Amann-Vesti
